# Substrate stiffness-dependent regulatory volume decrease and calcium signaling in chondrocytes: Substrate stiffness regulates chondrocyte volume and calcium signaling

**DOI:** 10.3724/abbs.2021008

**Published:** 2021-12-16

**Authors:** Min Zhang, Xiaoan Wu, Genlai Du, Weiyi Chen, Quanyou Zhang

**Affiliations:** 1.College of Biomedical Engineering Taiyuan University of Technology Taiyuan 030024 China; 2.Department of Physiology and Biophysics Miller School of Medicine University of Miami Miami FL 33136 USA; 3.Department of Cell Biology and Medical Genetics School of Basic Medical Science Shanxi Medical University Taiyuan 030001 China; 4.Department of Orthopaedics the Second Hospital of Shanxi Medical University Shanxi Key Laboratory of Bone and Soft Tissue Injury Repair Shanxi Medical University Taiyuan 030001 China

**Keywords:** substrate stiffness, regulatory volume decrease, viscoelasticity, calcium signaling, TRPV4

## Abstract

The pericellular matrix stiffness is strongly associated with its biochemical and structural changes during the aging and osteoarthritis progress of articular cartilage. However, how substrate stiffness modulates the chondrocyte regulatory volume decrease (RVD) and calcium signaling in chondrocytes remains unknown. This study aims to investigate the effects of substrate stiffness on the chondrocyte RVD and calcium signaling by recapitulating the physiologically relevant substrate stiffness. Our results showed that substrate stiffness induces completely different dynamical deformations between the cell swelling and recovering progresses. Chondrocytes swell faster on the soft substrate but recovers slower than the stiff substrate during the RVD response induced by the hypo-osmotic challenge. We found that stiff substrate enhances the cytosolic Ca ^2+^ oscillation of chondrocytes in the iso-osmotic medium. Furthermore, chondrocytes exhibit a distinctive cytosolic Ca ^2+^ oscillation during the RVD response. Soft substrate significantly improves the Ca ^2+^ oscillation in the cell swelling process whereas stiff substrate enhances the cytosolic Ca ^2+^ oscillation in the cell recovering process. Our work also suggests that the TRPV4 channel is involved in the chondrocyte sensing substrate stiffness by mediating Ca ^2+^ signaling in a stiffness-dependent manner. This helps to understand a previously unidentified relationship between substrate stiffness and RVD response under the hypo-osmotic challenge. A better understanding of substrate stiffness regulating chondrocyte volume and calcium signaling will aid the development of novel cell-instructive biomaterial to restore cellular functions.

## Introduction

Chondrocytes are responsible for the regulation of the fate of articular cartilage by synthesizing the structural components of the extracellular matrix as well as the matrix-degrading proteases [1]. The pericellular matrix (PCM) with defined mechanical properties surrounds each chondrocyte [2] and plays a critical role in regulating the mechanical microenvironment of chondrocytes [ 3, 4]. During the aging and osteoarthritis (OA) progress of articular cartilage, the mechanical properties (i.e. stiffness) of the PCM can markedly change between 1 kPa and 205 kPa [ 5– 8]. The PCM stiffness can be reduced by 30%–50% in the development of OA [9], which modulates the chondrocyte function-related spatial organization and biosynthetic activity [ 10, 11]. Many mechanosensation and mechanotransduction pathways in cartilage involve important mechanical sensitive ion channels [12]. The loss of PCM stiffness corresponds to the increased extent of cell swelling and calcium signaling mediated by transient receptor potential vanilloid channel 4 (TRPV4) in response to osmotic stress [ 13, 14]. Cell volume regulation is important to chondrocyte physiology. Under the hypo-osmotic stimulus, chondrocyte volume rapidly increases, followed by the active regulatory volume decrease (RVD) which exhibits an active volume recovery process [15]. However, how the change in the PCM stiffness influences the RVD and TRPV4-mediated calcium signaling in chondrocytes remains unknown.

Cell-supporting substrate stiffness, as a key physical factor of the cell microenvironment, is well recognized to regulate different cell functions and cell-to-cell communication [ 16, 17]. Substrate stiffness has been shown to modulate the chondrocyte morphology, phenotype and mechanical behavior [ 18, 19]. The RVD is crucial for many biophysical and biological responses of chondrocytes. Loss of chondrocyte RVD is associated with cell death and the progression of OA [ 20, 21]. Volume change affects chondrocyte phenotypic plasticity as well as the matrix metabolism [ 22, 23]. For example, the RVD-induced changes in cell shape can affect membrane transporter activity potentially mediated via calcium signaling. The sensing of matrix stiffness by chondrocytes is dependent on calcium signaling [24]. TRPV4 has been proposed as a mechanosensitive channel that is involved in regulating the mechanotransduction and RVD response of chondrocytes [ 25– 30]. Notably, TRPV4, as the primary regulator of calcium signaling, is assumed to be involved in cell sensing substrate stiffness by locally altering the Ca ^2+^ permeability [ 31, 32]. Since calcium signaling plays an important role in multiple signaling pathways and mechanotransduction in chondrocytes [ 33, 34], substrate stiffness may modulate the chondrocyte mechanotransduction via the TRPV4-mediated Ca ^2+^ signaling, which eventually affects cell function [16].

Collectively, these findings highlight the potential role of substrate stiffness in regulating the RVD response and TRPV4-mediated calcium signaling in chondrocytes. A previous study suggested that PCM stiffness affects the chondrocyte volume swelling and TRPV4-mediated Ca ^2+^ signaling [14]. However, these studies mainly focus on the swelling process and lack the dynamic recovering process of chondrocytes. To date, neither theoretical nor experimental studies have been carried out to explore the role of substrate stiffness in the RVD response and TRPV4-mediated Ca ^2+^ oscillations during the chondrocyte swelling and recovering processes.

In the present study, we hypothesized that: (I) substrate stiffness modulates the mechanical properties of chondrocytes, resulting in the substrate stiffness-dependent RVD response, and (II) TRPV4 channels are involved in chondrocytes sensing substrate stiffness by modulating cytosolic Ca ^2+^ signaling. To test these hypotheses, polydimethylsiloxane (PDMS) was utilized to engineer cell-supporting substrate with physiologically relevant stiffness. The mechanical properties of chondrocytes were evaluated by atomic force microscopy (AFM). Ca ^2+^ oscillation of chondrocytes cultured on variable stiffness substrates was quantitatively characterized.

## Materials and Methods

### Preparation of substrates with different stiffness

PDMS substrate stiffness was formulated to mimic the physiologically-related PCM elasticity of stiff (2.2±0.3 MPa), medium (46.5±5.8 kPa) or soft (2.1±0.2 kPa), respectively [19]. Briefly, PDMS curing agent (Sylgard184; Dow Corning Corp, St Louis, USA) was mixed with a base agent in a mass ratio of 1:10 (stiff), 1:50 (medium) or 1:70 (soft), respectively. The mixture was poured into a 35-mm petri dish to create a 1-mm thick film and then cured at 70°C for 6 h. After curing, these PDMS substrates were placed in an oxygen plasma cleaner (SBC-12; KYKY, Beijing, China) for surface oxidation. The stiffness of PDMS substrate (elastic modulus) was determined using the indentation method with the ElectroForceH3100 test instrument (Bose, Shanghai, China) as previously described [35]. Before cells were seeded, substrates with different stiffness were coated with 0.02 mg/mL rat type I collagen (Shengyou Biotechnology, Hangzhou, China) at 4°C overnight and sterilized by UV irradiation for 45 min as previously described [19].

### Isolation of primary chondrocytes

A total of 24 C57BL/6 mice (5–6 days old) purchased from Shanxi Medical University (Taiyuan, China) were used in this study. All procedures were approved by the Animal Ethics Committee of Taiyuan University of Technology and the animal experiments were conducted under the International Guidelines and Standards on Animal Welfare according to the Animal Research: Reporting In Vivo Experiments (ARRIVE) Guidelines [36]. Mice were sacrificed under general anesthesia, and the femoral condyles and tibial plateau were isolated from the hind limbs. The pieces of cartilage were incubated in the collagenase D digestion solution (3 mg/mL) for 45 min at 37°C in an incubator with 5% CO _2_. Then the cartilage pieces were placed in a new Petri dish with collagenase D solution (0.5 mg/mL) and incubated overnight at 37°C as previously described [37]. Then cells were isolated by filtering through a 40-μm cell strainer (BD-Falcon, Franklin Lakes, USA). The cell suspension was washed twice with fresh Dulbecco’s modified Eagle’s medium (DMEM; Sigma, St Louis, USA). Cells were resuspended in DMEM supplemented with 10% fetal calf serum, 1 g/mL glucose, 2 mM GIn and 1% penicillin/streptomycin, an iso-osmotic medium with a final osmolarity of 320 mOsm. Cells were grown on substrates of different stiffness at 37°C with 5% CO _2_. In the RVD and AFM experiments, chondrocytes cultured on different substrate stiffness were exposed to hypo-osmotic medium. All experiments were performed at 37°C under controlled humidity in a custom-built equipment within 1.5 h unless otherwise stated.

### Measurement of RVD response

The chondrocytes were cultured for 24 h in the iso-osmotic medium before exposure to the hypo-osmotic medium. After the hypo-osmotic challenge, chondrocyte RVD response suggests that cells initially swell passively within a few minutes, and then cell volume reduces towards the initial value. Cells are considered to show RVD response if the cell diameter is reduced by at least 1 μm during the imaging period [38]. The whole process of RVD response was tracked. Bright-field sequential time-lapse images of chondrocytes on substrates of different stiffness were obtained every 10 s for 45 min under a microscope (FV1000; Olympus, Tokyo, Japan) with a 40× oil immersion objective lens. The diameter (d) of a single cell was quantitatively calculated from the mean of the two orthogonal diameters using ImageJ software (Version 1.50b; National Institutes of Health, Bethesda, USA). Then, the following parameters were analyzed. The swelling time, *T*_S_, is the time that takes for the cell to swell until it reaches its maximum cell diameter during the hypo-osmotic challenge. The recovering time, *T*_R_, is the time that takes for the cell to recover from its maximum diameter until its diameter no longer changes. *T*_Res_, is the time of the whole RVD process which equals the sum of *T*_S_ and *T*_R_. The cell diameter rate, *V*_d_, is the cell diameter rate over time during the RVD response.

### Atomic force microscopy (AFM)

In AFM experiments, we only focused on the changes in the mechanical properties of chondrocytes on substrates of different stiffness during cell swelling. The mechanical properties of a single chondrocyte were measured with the NTEGRA Solaris atomic force microscope (NT-MDT, Moscow, Russia) combined with a phase-contrast microscope (IX-70; Olympus). A gold-coated, 5-μm diameter, spherical tip cantilever with a spring constant of 0.05 N/m (Novascan Technologies, Inc., Boone, USA) was used for the indentation and stress-relaxation experiments. Indentations were applied with a force trigger of 2.5 nN. An approach velocity of 6.53 μm/s was used, followed by a 100 s relaxation period. A constant displacement of about 2 μm indentation was held for all the stress-relaxation experiments. The elastic and viscoelastic parameters of chondrocyte were determined according to a modified Hertz equation (Eq.1) and a stress relaxation model of viscoelastic solid (Eq.2), respectively [39].

**Table:** 

$$\begin{array}{lr} F\left(\delta \right)=\frac{4R^{\frac{\text{1}}{\text{2}}}E_{elastic}}{3\left(1-\nu^{2}\right)}\delta^{\frac{\text{3}}{\text{2}}}C & \text{(1)} \end{array}$$

**Table:** 

$$\begin{array}{lr} \begin{array}{l} \underset{}{F\left(t\right)=\frac{4R^{\frac{\text{1}}{\text{2}}}\delta_{0}^{\frac{3}{2}}E_{R}}{3\left(1-\nu \right)}\left(1+\frac{\tau_{\sigma }-\tau_{\varepsilon }}{\tau_{\varepsilon }}e^{-\frac{t}{\tau_{\varepsilon }}}\right)C}\\ E_{0}=E_{R}\left(1+\frac{\tau_{\sigma }-\tau_{\varepsilon }}{\tau_{\varepsilon }}\right)，\mu =E_{R}\left(\tau_{\sigma }-\tau_{\varepsilon }\right) \end{array}\} & \text{(2)} \end{array}$$

where *F* is the applied force, *R*is the relative radius, ν is the Poisson’s ratio which is assumed to be 0.5 [39], *C* is a constant depending only on the indentation during stress relaxation. The elastic modulus, *E*_elastic_, is determined by fitting the indentation approach curves with Hertz equation (Eq.1). *δ* is the indentation depth. *E*_R_ and *E*_0_ are instantaneous modulus and relaxed modulus, respectively. τ _σ_ represents the time taken for creep in response to a constant stress. τ _ε_ represents the time taken for stress relaxation in response to a constant strain. *μ* is the apparent viscosity.

### Western blot analysis and immunohistochemistry

Total protein extraction was carried out using RIPA lysis buffer (Roche, Basel, Switzerland). Samples were mixed with 4×SDS loading buffer and denatured at 90°C for 5 min. The proteins were separated by SDS-PAGE, and then transferred onto PVDF membranes (Millipore, Billerica, USA). After being blocked and washed, membranes were incubated overnight at 4°C with primary antibodies against TRPV4 (1:500 dilution; Alomone Laboratories, Jerusalem, Israel), followed by incubation with horseradish peroxidase-conjugated goat anti-rabbit IgG (1:8000 dilution; Tiangen, Beijing, China) for 1 h at room temperature (RT). Bound antibodies were detected using ECL western blot enhanced chemiluminescence kit (Thermo Pierce, Waltham, USA).

Samples were fixed with 4% paraformaldehyde for 10 min, permeabilized by 0.1% Triton X-100 in PBS for 10 min, and blocked by 5% BSA for 30 min. To visualize the TRPV4 channel expression, fixed cells were incubated with primary antibody (1:400 dilution; Alomone Laboratories) at 4ºC overnight. Then AlexaFluor-555-conjugated secondary antibody (Molecular Probes, San Jose, USA) was added at a dilution of 1:100 on the next day. Finally, cells were treated with 5 mg/mL 4′,6-diamidino-2-phenylindole (DAPI; Sigma) for 5 min to stain the nuclei and the cell images were captured by using an excitation wavelength of 488 nm and a 40× oil immersion objective lens on a confocal microscope.

### Semi-quantitative reverse transcription polymerase chain reaction

Extracted RNA was reverse-transcribed into cDNA using the Goscript ^TM^ Reverse Transcriptase System (Promega, Madison, USA) according to the manufacturer’s instructions. Semi-quantitative PCR was performed using a PCR kit (Tiangen, Beijing, China) on a thermo-cycler (Bio-Rad, Hercules, USA). PCR reactions were performed in a total volume of 25 μL containing 1 μL cDNA. Primers of the TRPV4 channel were designed for semi-quantitative PCR. All specific primers for the TRPV4 channel were designed based on Genbank mouse sequences. Specific primer sequences are as follows: TRPV4 channel, sense: 5′-TACGACCTGCTGCTTCTCAA-3′, antisense: 5′-TCCTCATCTGTCACCTCACG-3′; and GAPDH, sense: 5′-GGTGAAGGTCGGTGTGAACG-3′, antisense: 5′-CTCGCTCCTGGAAGATGGTG-3′. The cDNA product was PCR amplified using specific primers for the TRPV4 channel. The PCR annealing temperature for each primer pair was optimized using a Master Cycler Gradient Thermocycler (Eppendorf, Hamburg, Germany). PCR reaction consisted of 30 s denaturation at 94°C, 30 s for annealing at 58–64°C, 30 s for elongation at 72°C, and 30 amplification cycles. PCR products (15 μL) were separated by electrophoresis on a 1% agarose gel in Tris-borate/EDTA buffer. The quantification of semi-quantitative PCR was performed by using the optical density (OD) method.

### Calcium imaging

Chondrocytes on substrates of different stiffness were loaded with Fluo-4 AM (Life Technology, Boston, USA) in pure DMEM buffer for 35 min at 37°C before imaging. To modulate the TRPV4 channel activity, 10 μM GSK205 or 1 μM 4αPDD (Millipore) was added to the bath solution. GSK205 is a TRPV4 selective antagonist, while 4αPDD is a TRPV4 selective agonist. Ca ^2+^ signaling imaging of chondrocytes was recorded in response to substrates of different stiffness and hypo-osmotic challenge on a fluorescence confocal laser scanning microscope. Time-series images of baseline Ca ^2+^ oscillations were recorded every 3 s for a total of 15 min and fluorescence was analyzed by using the ImageJ software. The difference (Δ *F*) between the mean fluorescence measured in a given region of interest (ROI) and the corresponding control value for each ROI ( *F*_0_) was expressed as the fraction of the control (Δ *F*/ *F*_0_) and then plotted as a function of time. In the RVD experiments, the cell diameter change and the chondrocyte Ca ^2+^ oscillations were simultaneously monitored. Time-series images of Ca ^2+^oscillations were recorded every 3 s during the whole process of chondrocyte RVD swelling and recovering. A cell was defined as responsive if it showed a calcium peak with a magnitude four times higher than its maximum fluctuation along the baseline [40].

### Statistical analysis

All data are presented as the mean±standard deviation. Statistical difference between the groups was estimated using one-way analysis of variance (ANOVA) followed by a Tukey post hoc test using Origin (OriginLab, Northampton, USA). *P* values less than 0.05 are considered to be statistically significant.

## Results

### Substrate stiffness affects the RVD response of chondrocytes

**Figure 1:**
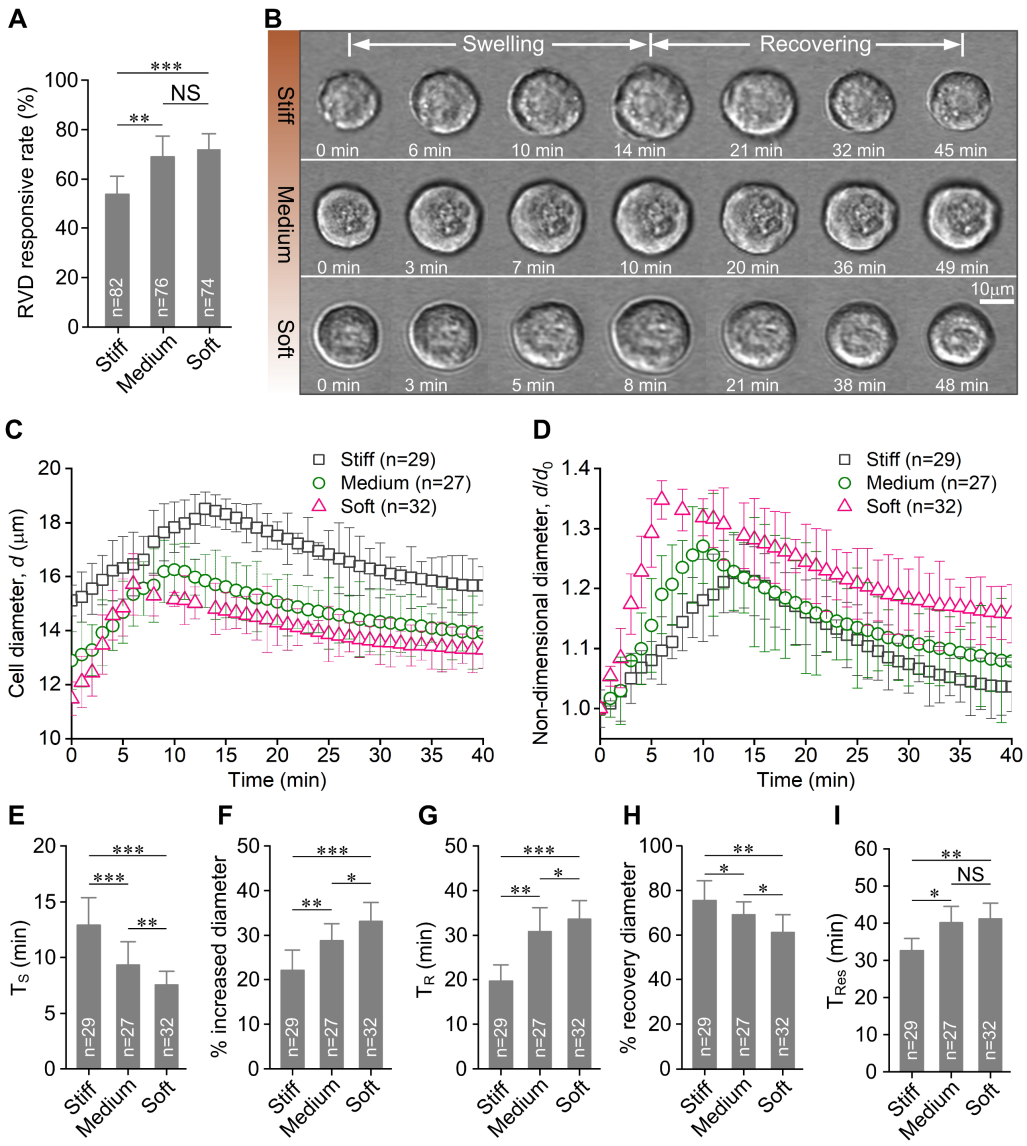
Figure1 **Substrate stiffness affects the hypo-osmotic challenge-induced RVD response of chondrocytes**(A) The percentage of RVD response in chondrocytes on the stiff substrate (55%±6%) was significantly lower than that on the medium (68%±6%) and soft substrate (72%±8%), respectively. The analysis of RVD responsive rate in 82 chondrocytes on the stiff substrate, 76 chondrocytes on the medium substrate, and 74 chondrocytes on the soft substrate. Each group is from 10 different experiments. (B) Representative time lapse recordings of the RVD response in three chondrocytes from the stiff, medium and soft substrate, respectively. (C) The diameter (d) changes of chondrocytes on different substrates during the complete RVD response. (D) The non-dimensional diameter (d/d0) change of chondrocytes on different substrates during the RVD response. The trend of d/d0 change with time is consistent with Figure1C. (E) The stiff substrate significantly increased the mean swelling time (TS) of chondrocytes during cell swelling. (F) The percentage increase of chondrocyte diameter was increased significantly with the decreasing substrate stiffness during cell swelling. (G) The mean recovery time (TR) of chondrocyte on the soft substrate was dramatically prolonged during the cell recovering. (H) The percentage of chondrocyte recovery diameter was increased with the increasing substrate stiffness. (I) The mean RVD responding time (TRes) of chondrocyte on the soft substrate was significantly longer than that on the stiff substrate. The swelling and recovering processes of chondrocytes significantly depend on the substrate stiffness during the RVD response. Statistical significance was analyzed by one-way ANOVA and post hoc Tukey’s test. NS, not significant (P>0.05). *P< 0.05, **P<0.01, and ***P<0.001.

Our results showed that the RVD response of chondrocytes was intimately related to substrate stiffness. The soft substrate markedly increased the percentage of RVD response in chondrocytes ( *P*<0.001; Figure 1A). The substrate stiffness caused distinctive swelling and recovering behaviors of chondrocytes ( Figure 1B). The change of cell diameter *d*depended on the substrate stiffness during the RVD response ( Figure 1C). The trend of non-dimensional diameter *d*/ *d*_0_ ( *d*_0_ is the initial cell diameter) over time remained the same with Figure 1C, and it was independent of the cell’s initial diameter ( Figure 1D). We also found that stiff substrate significantly prolonged the cell swelling time *T*_S_ ( *P*<0.001; Supplementary Table S1 and Figure 1E), and soft substrate significantly increased the percentage increase of diameter in chondrocytes during cell swelling ( *P*<0.001; Figure 1F). In contrast, during cell recovery, the recovery time *T*_R_ in chondrocytes on the stiff substrate was significantly shorter than that on the soft substrate ( *P*<0.05; Figure 1G). Furthermore, the stiff substrate enhanced the capacity of cell diameter recovery during the cell recovering ( *P*<0.01; Figure 1H). Interestingly, the trend of the swelling and recovering time was opposite to that of the percentage increase of diameter in chondrocytes during cell RVD response. Lastly, the soft substrate significantly increased the RVD responding time *T*_Res_ of chondrocytes ( *P*<0.01; Figure 1I).

**Figure 2:**
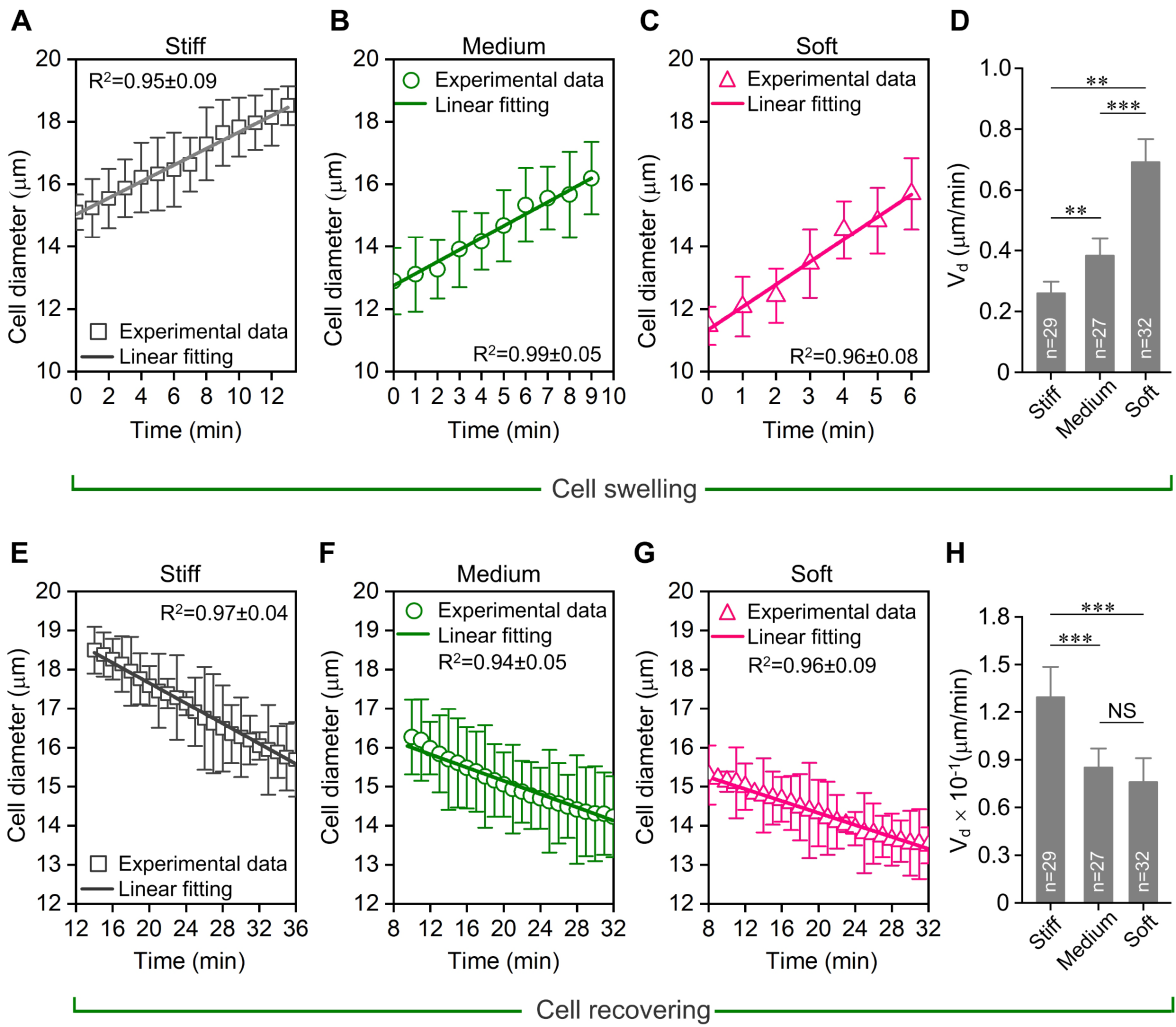
Figure2 **Quantification of chondrocyte diameter rate during the hypo-osmotic challenge-induced RVD response**(A-C) The linearly fitting of cell diameter vs time curves on substrates with different stiffness during cell swelling. The diameter rate, Vd is the slope of the line. The cell diameter rate in the stiff, medium and soft substrate are Vd=0.26±0.02 μm/min, Vd=0.38±0.03 μm/min and Vd=0.72±0.04 μm/min, respectively. (D) The soft substrate significantly increased the cell diameter rate during cell swelling. (E–G) The linearly fitting of cell diameter vs time curves on substrates of varying stiffness during the cell recovering. The diameter change curve from the maximum diameter to the state of equilibrium was used to fit. The cell diameter change rate in stiff, medium and soft substrate are Vd=0.13±0.02 μm/min, Vd=0.09±0.01 μm/min and Vd=0.08±0.01 μm/min, respectively. (H) The stiff substrate enhanced the diameter rate of chondrocytes during the cell recovering process. A higher coefficient of determination (R2) indicates a better linear match. Statistical significance was analyzed by one-way ANOVA and post hoc Tukey’s test. NS, not significant (P>0.05). **P<0.01 and ***P<0.001.

The cell diameter rate was obtained by fitting the cell diameter vs time curves from substrates of different stiffness. The cell diameter vs time curves from all substrates during cell swelling exhibited a linear relationship ( Figure 2A–C). Moreover, the soft substrate markedly increased the cell diameter rate during cell swelling caused by the hypo-osmotic medium ( *P*<0.001; Figure 2D). Then, we fitted the approximately linear part of cell diameter vs time curves during the cell recovering ( Figure 2E–G). However, stiff substrate significantly increased the recovering cell diameter rate within about 20 min of initial recovery ( *P*<0.001; Figure 2H). Our results indicated for the first time that hypo-osmotic challenge-induced chondrocyte deformation is dependent on substrate stiffness.

### Stiff substrate enhances the mechanical properties of chondrocytes during cell swelling

**Figure 3:**
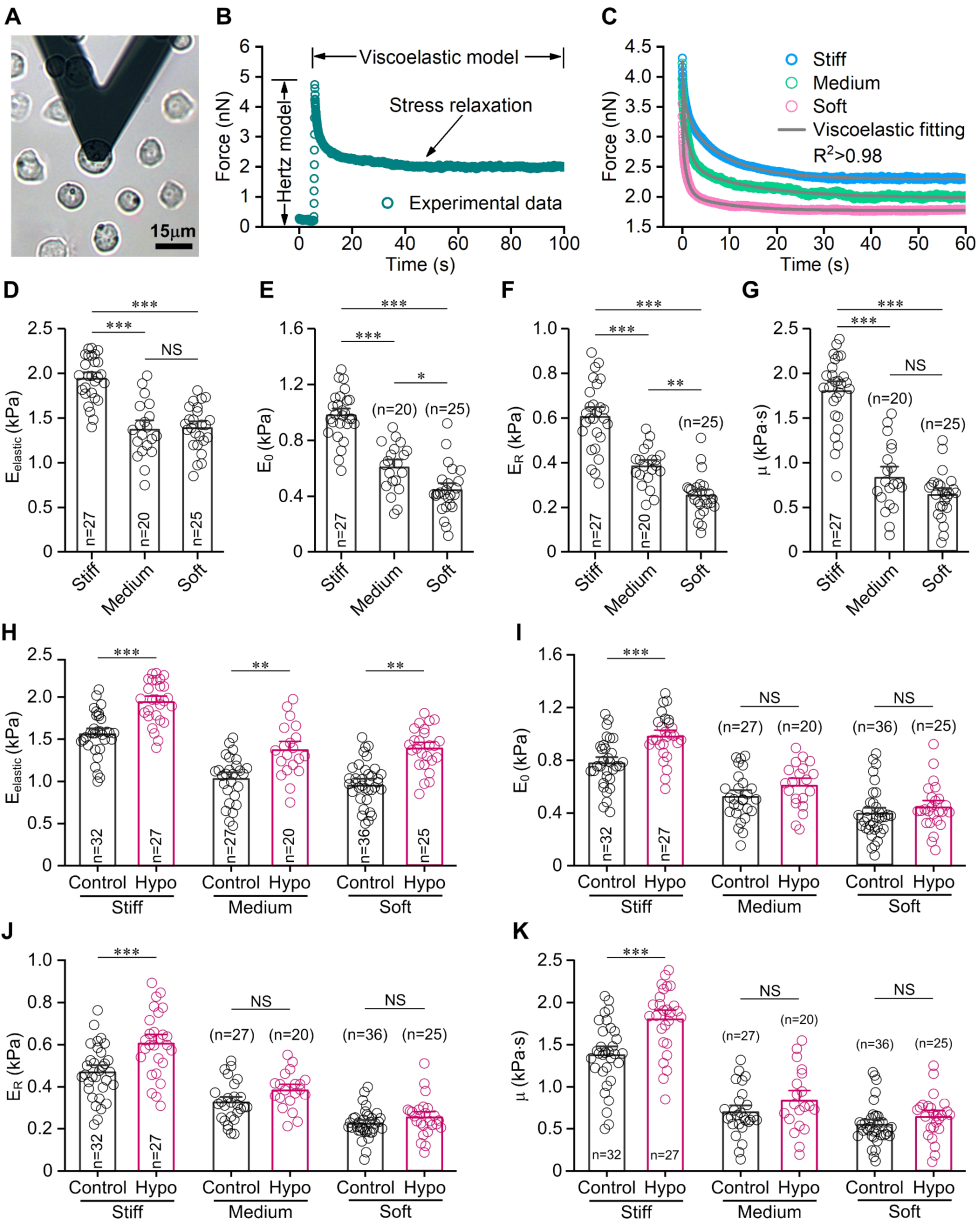
Figure3 **AFM indentation and the analysis of mechanical properties of chondrocyte on substrates with different stiffness during cell swelling in 180 mOsm medium All cells were seeded at a density of 2×10 ^4^ cells/cm ^2^ in the AFM experiment. **(A) Schematic illustration of the AFM measurement. Chondrocytes maintained a spherical shape after 24 h of cell seeding onto substrates. The AFM probe was positioned over the perinuclear region of a single chondrocyte in AFM experiments. Only chondrocytes with the RVD response were chosen for mechanical analysis. AFM experiments were performed 3 min after exposure to the hypo-osmotic challenge. (B) Representative elastic and time-dependent stress relaxation response of single chondrocyte in AFM experiments. Chondrocyte usually exhibits the typical elastic response and subsequently stress-relaxation process. The Eelastic of a chondrocyte was extracted from the initial indentation according to the Hertz model. The viscoelastic properties were extracted by fitting the stress-relaxation curve. (C) Representative stress-relaxation curves and theoretical fitting of chondrocytes from different substrates during cell swelling, respectively. A higher coefficient of determination (R2) indicates a better match between the stress relaxation model and experimental data. (D-G) Comparison of mechanical parameters of chondrocytes on different substrates during cell swelling. The stiff substrate markedly increased the mechanical parameters (Eelastic, ER, E0 and μ) of chondrocytes. (H-K) Comparison of mechanical parameters of chondrocytes on substrates in the iso-osmotic (control group) and hypo-osmotic medium. NS, not significant (P>0.05). *P<0.05, **P<0.01, ***P<0.001 determined by one-way ANOVA and post hoc Tukey’s test.

AFM was used to measure the mechanical properties of chondrocytes on substrates with different stiffness in the iso-osmotic and hypo-osmotic media ( Figure 3A). When the constant indentation displacement of 2 μm was held on chondrocytes, chondrocytes first exhibited typical elastic response consistent with the Hertz model and then exhibited stress relaxation consistent with the viscoelastic theoretical model ( Figure 3B). Theoretical model fitting of all experimental data exhibited excellent consistency for elastic and viscoelastic equations respectively ( Figure 3C). Our results indicated that stiff substrate enhances the elastic ( *E*_elastic_) and viscoelastic parameters ( *E*_R_, *E*_0_ and *μ*) of chondrocytes during cell swelling in the hypo-osmotic medium ( *P*<0.001; Table 1 and Figure 3D–G).

Then we compared the mechanical parameters of chondrocytes on substrates with different stiffness between the iso-osmotic ( Supplementary Table S2) and hypo-osmotic medium. Our results showed that, compared to the iso-osmotic medium, chondrocytes on the stiff substrate during cell swelling in the hypo-osmotic medium showed higher elastic and viscoelastic parameters including *E*_elastic_, *E*_R_, *E*_0_ and *μ* ( *P*<0.001; Figure 3H–K). Moreover, the mean percentage increase in the elastic modulus *E*_elastic_ of chondrocytes was higher on the soft substrate than on the stiff substrate during cell swelling. However, in response to the hypo-osmotic medium, the mean percentage increases in the viscoelastic parameters ( *E*_R_, *E*_0_ and *μ*) of chondrocytes were higher on the stiff substrate than on the soft substrate ( Supplementary Table S3). These results suggested that substrate stiffness regulates the dynamic elastic and viscoelastic properties of chondrocytes during the hypo-osmotic challenge.

### Stiff substrate enhances cytosolic Ca ^2+^ oscillation of chondrocytes in iso-osmotic medium

**Figure 4:**
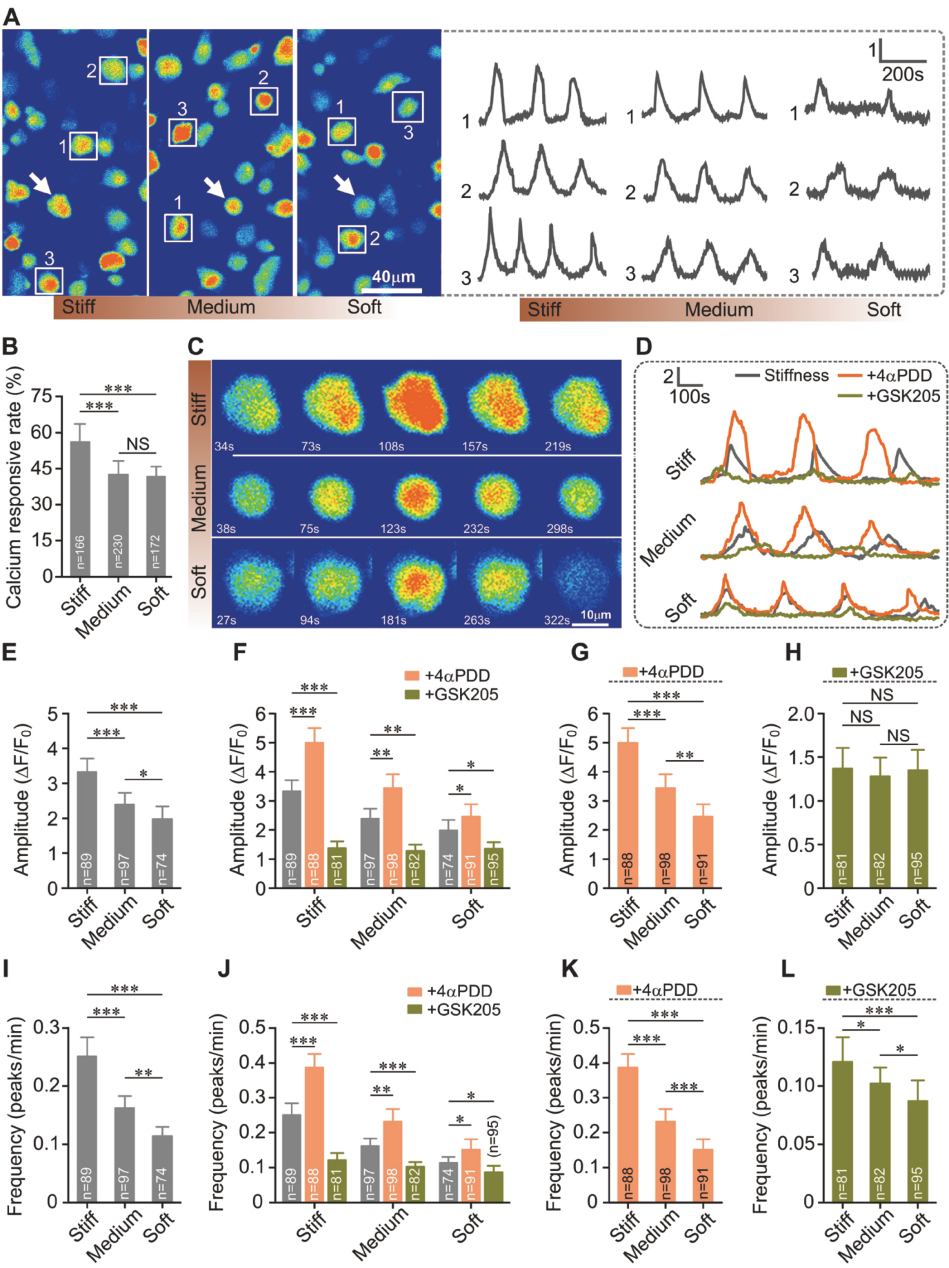
Figure4 **The effects of substrate stiffness on cytosolic Ca ^2+^ oscillations in chondrocytes in the iso-osmotic medium **(A) Representative cell images showing Ca2+ fluorescence in chondrocytes on different substrates, as marked with square and white arrows in figures. Time elapse recordings of Ca2+ oscillations of three chondrocytes on the stiff, medium and soft substrates, respectively, as denoted by the square in corresponding Figure4A (right). (B) The percentage of Ca2+ oscillation in chondrocytes was significantly increased on the stiff substrates. The calcium responsive rate of chondrocytes on the stiff, medium and soft substrate in the iso-osmotic medium was 56%±8%, 43%±6% and 42%±4%, respectively. (C) Representative chondrocytes showing cytosolic Ca2+ oscillations from the stiff, medium and soft substrate, respectively, as marked with white arrows in Figure4A. (D) Representative time lapse recordings of Ca2+ oscillations of chondrocytes on the stiff, medium and soft substrates treated by 4αPDD and GSK205. (E) The amplitude of Ca2+ oscillations in chondrocytes was significantly higher on the stiff substrate than on the soft substrates. (F) Comparison of the amplitude of Ca2+ oscillations in chondrocytes on different substrates with or without pharmacological treatment. (G) The amplitude of Ca2+ oscillations in chondrocytes activated by 4αPDD was significantly higher on the stiff substrate than on the soft substrate. (H) The amplitude of Ca2+ oscillations in chondrocytes on substrates with different stiffness inhibited by GSK205 showed no significant difference. (I) The frequency of Ca2+ oscillations in chondrocytes was significantly higher on the stiff substrate than on the soft substrate. (J) Comparison of the frequency of Ca2+ oscillations in chondrocytes on substrates of different stiffness with or without pharmacological treatment. (K) The frequency of Ca2+ oscillations in chondrocytes activated by 4αPDD was significantly higher on the stiff substrate than on the soft substrate. (L) The frequency of Ca2+ oscillations in chondrocytes inhibited by GSK205 was significantly higher on the stiff substrate than on the soft substrate. The cytosolic Ca2+ oscillations in chondrocytes were mediated by TRPV4 in a substrate stiffness-dependent manner. NS, not significant (P>0.05). *P<0.05, **P<0.01, ***P<0.001 determined by one-way ANOVA and post hoc Tukey’s test.

Chondrocytes on substrates with different stiffness showed cytosolic Ca ^2+^ oscillations in the iso-osmotic medium ( Figure 4A). Stiff substrate showed the greatest percentage of Ca ^2+^ oscillations of chondrocytes ( *P*<0.001; Figure 4B). We recorded the amplitude and frequency of Ca ^2+^ oscillation in chondrocytes ( Figure 4C,D). Our results showed that stiff substrate markedly enhanced both the amplitude and frequency of Ca ^2+^ oscillations in chondrocytes ( *P*<0.001; Figure 4E,I). To test if TRPV4 channels are involved in substrate stiffness-mediated Ca ^2+^ signaling in chondrocytes, we applied 4αPDD and GSK205 respectively when recording Ca ^2+^ oscillations. The results showed that 4αPDD significantly enhanced the calcium responsive rate, amplitude and frequency of Ca ^2+^ oscillations in chondrocytes on all substrates of different stiffness, while GSK205 weakened those Ca ^2+^ effects ( *P*<0.01; Figure 4D,F-H,J-L and Supplementary Table S4). In addition, when treated with 4αPDD, chondrocytes on the stiff substrate showed the highest amplitude and frequency of Ca ^2+^ oscillations ( *P*<0.001; Figure 4G,K). Chondrocytes on the stiff substrate also showed a bigger percentage increase of amplitude and frequency of Ca ^2+^ oscillations than those on the soft substrate ( Supplementary Table S4). On the other hand, when treated with the TRPV4 inhibitor GSK205, chondrocytes on substrates of different stiffness showed no significant difference in the amplitude of Ca ^2+^ oscillations ( *P*>0.25; Figure 4H), though chondrocytes on the stiff substrate exhibited a higher frequency of Ca ^2+^ oscillations ( *P*<0.05; Figure 4L). Chondrocytes showed a bigger percentage decrease of amplitude and frequency of Ca ^2+^ oscillations on the stiff substrate than on the soft substrate with the application of GSK205 ( Supplementary Table S5). Taken together, these results suggested that the mechanosensitive TRPV4 channel is involved in chondrocytes sensing substrate stiffness and mediating the calcium signaling.

### Substrate stiffness affects the hypo-osmotic challenge-induced Ca ^2+^ oscillations in chondrocytes

**Figure 5:**
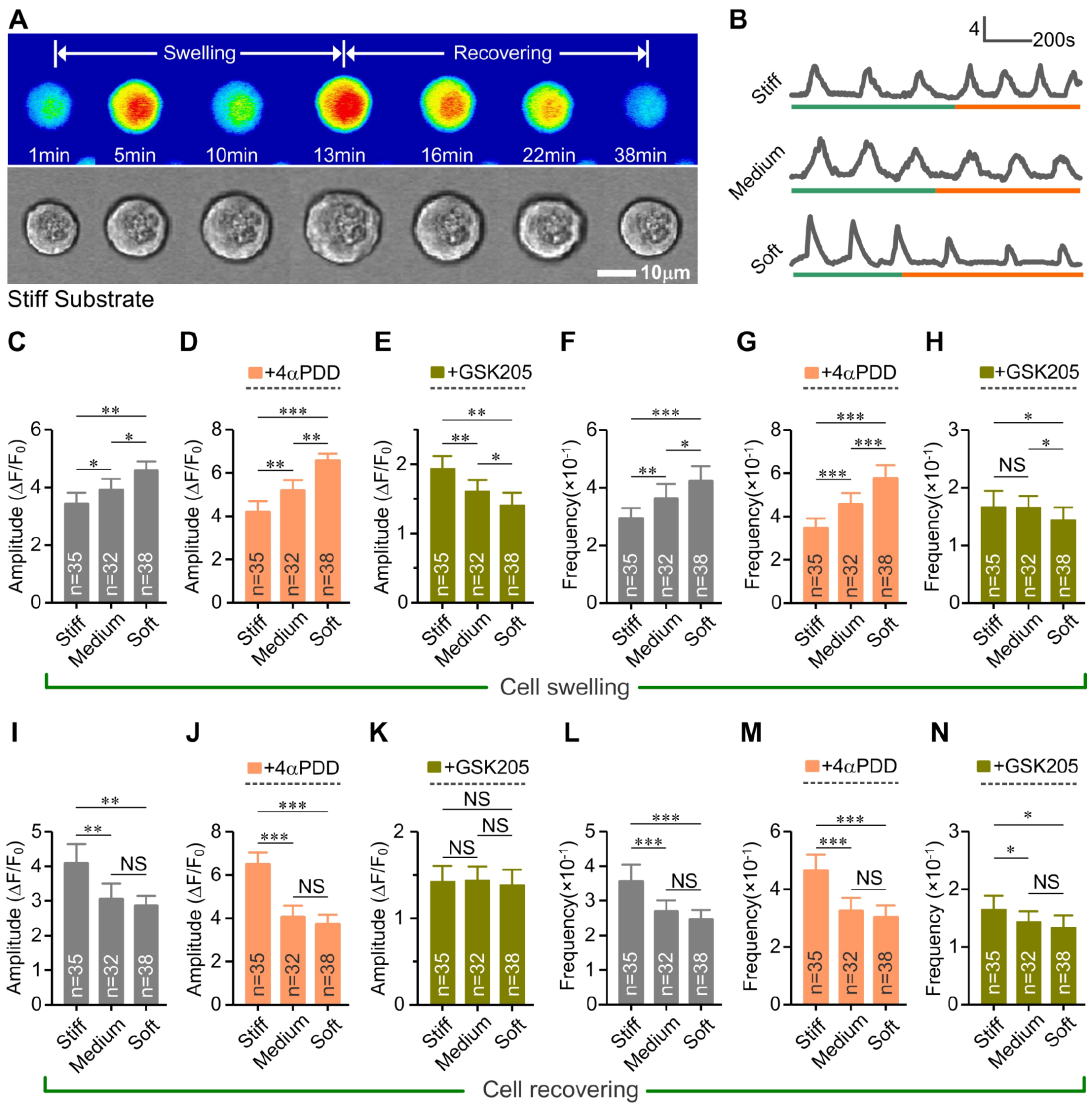
Figure5 **Quantitative analysis of Ca ^2+^ oscillations in chondrocytes on substrates with different stiffness in the hypo-osmotic medium **(A) Representative single-cell images showing Ca2+ fluorescence in chondrocyte on the stiff substrate during the RVD response. (B) Representative time-lapse recordings of Ca2+ oscillations in chondrocytes on the stiff, medium and soft substrate in the cell RVD response, respectively. The green lines represent the cell swelling process and the red lines represent the cell recovering process. (C-H) Summary of the amplitude and frequency of cytosolic Ca2+ oscillations in chondrocytes on substrates with different stiffness with or without 4αPDD or GSK205 during cell swelling. The amplitude and frequency of cytosolic Ca2+ oscillations in chondrocytes were significantly higher on the soft substrate than on the stiff substrate during cell swelling. (I-N) Summary of the amplitude and frequency of cytosolic Ca2+ oscillations in chondrocytes on substrates with different stiffness with or without 4αPDD or GSK205 during cell recovering. The amplitude and frequency of cytosolic Ca2+ oscillations in chondrocytes were significantly higher on the stiff substrate than on the softer substrate. The spontaneous cytosolic Ca2+ oscillations in chondrocytes were mediated by TRPV4 in a substrate stiffness-dependent manner during the cell RVD response. The numbers shown in parenthesis indicate the number of cells analyzed per substrate stiffness. NS, not significant (P>0.05). *P< 0.05, **P<0.01, ***P<0.001 determined by one-way ANOVA and post hoc Tukey’s test.

Only chondrocytes that exhibited both the RVD response and Ca ^2+^ oscillations were used in this study. Our results showed that soft substrates significantly increased the percentage of chondrocytes that exhibited both the RVD response and Ca ^2+^ oscillations (42%±5% and 39%±4%, respectively) than the stiff substrate (31%±5%; *P*<0.01). Then, we quantified the amplitude and frequency of Ca ^2+^ oscillation in chondrocytes on substrates during cell swelling and recovering ( Figure 5A,B). The results showed that soft substrate induced a significantly higher percentage of Ca ^2+^ oscillations (58%±7%) in chondrocytes than medium (77%±8%) and stiff substrates (79%±8%) during the hypo-osmotic challenge ( *P*<0.001). 4αPDD significantly enhanced but GSK205 significantly weakened the calcium responsive rate of Ca ^2+^ oscillations in chondrocytes on different substrates ( *P*<0.01; Supplementary Table S6). During cell swelling, the soft substrate enhanced both the amplitude and frequency of Ca ^2+^ oscillations ( *P*<0.01; Figure 5C,F). After treatment with 4αPDD, both the amplitude and frequency of Ca ^2+^ oscillations in chondrocytes were significantly increased on the soft substrate ( *P*<0.005; Figure 5D,G), while after treatment with GSK205, the amplitude and frequency of Ca ^2+^ oscillations were significantly increased on the stiff substrate ( *P*<0.05; Figure 5E,H). Moreover, the relative percentage increase of Ca ^2+^ oscillations (amplitude and frequency) in chondrocytes treated with 4αPDD was higher on the soft substrate than on the stiff substrate during cell swelling. On the other hand, the decrease of Ca ^2+^ oscillations induced by GSK205 was also higher on the soft substrate than on the stiff substrate ( Supplementary Table S7).

During the cell recovering process, the stiff substrate enhanced both the amplitude and frequency of Ca ^2+^ oscillations in chondrocytes ( *P*<0.001; Figure 5I,L). After treatment with 4αPDD, both the amplitude and frequency of Ca ^2+^ oscillations in chondrocytes were significantly increased on the stiff substrate ( *P*<0.01; Figure 5J,M). After treatment with GSK205, the amplitude of Ca ^2+^ oscillations in chondrocytes showed no significant difference between substrates with different stiffness ( *P*>0.05; Figure 5K), but the frequency of Ca ^2+^ oscillations in chondrocytes was significantly higher on the stiff substrate than on the soft substrate ( *P*<0.05; Figure 5N). Moreover, after treatment with 4αPDD or GSK205, stiff substrate induced a higher percentage increase or decrease in Ca ^2+^ oscillation in chondrocytes than the soft substrate ( Supplementary Table S8), respectively. Our results, therefore, suggested that the TRPV4 channel is involved in chondrocyte RVD response, exhibiting a completely different substrate stiffness-dependent behavior of Ca ^2+^ oscillations between the cell swelling and recovering progress.

### Stiff substrate up-regulates the TRPV4 expression in chondrocytes in the iso-osmotic medium

**Figure 6:**
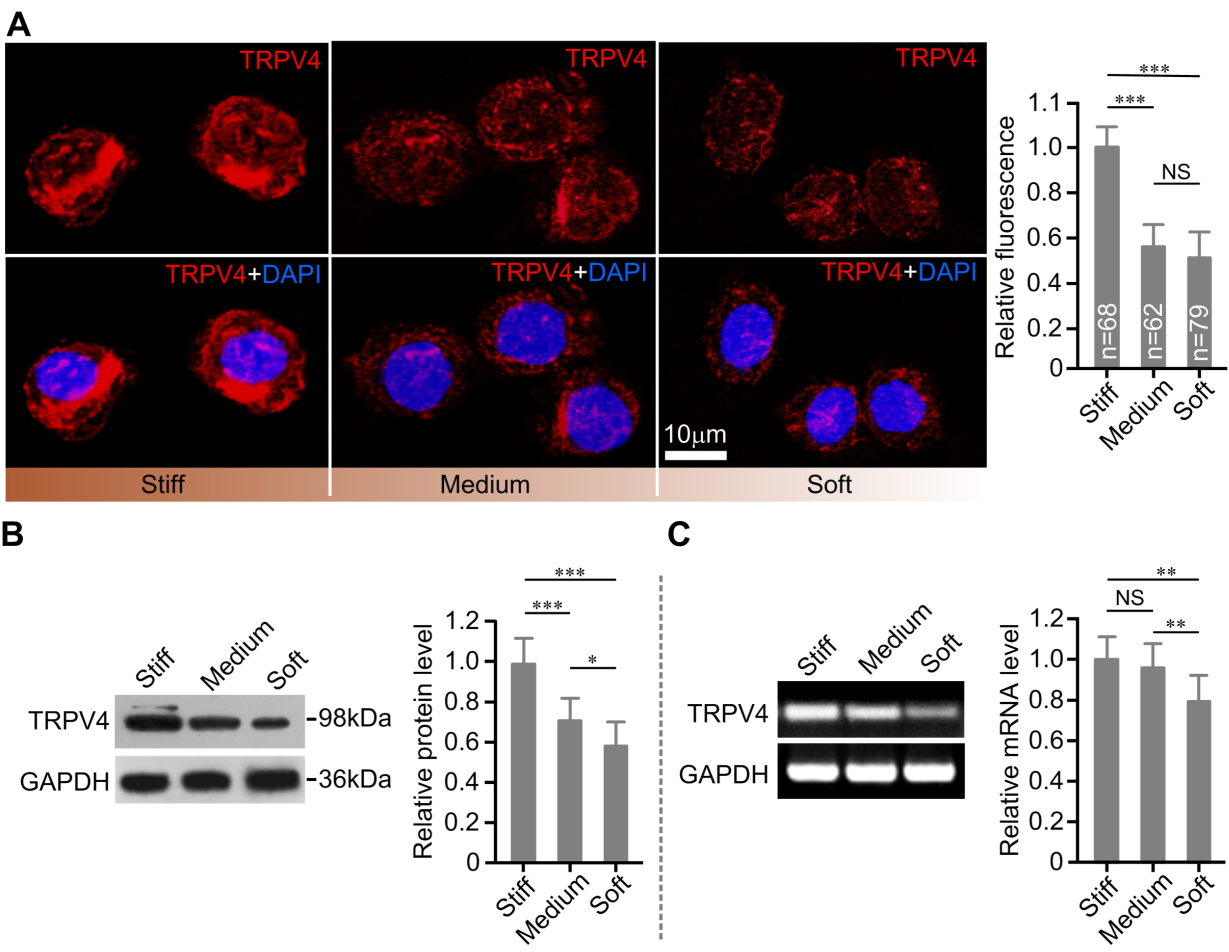
Figure6 **The effects of substrate stiffness on the expression level of TRPV4 channels**(A) Immunohistochemistry staining of TRPV4 channels (red, top) and merged with nuclei (blue, bottom) of chondrocytes on stiff, medium and soft substrate, respectively. Quantitative analysis of TRPV4 fluorescence intensity indicated that the expression of the TRPV4 in chondrocytes is higher on the stiff substrate than on the soft substrate (right panel). Statistical results from three independent experiments. (B) Western blot analysis and quantification of TRPV4 channel protein expression of chondrocytes on different substrates. The stiff substrate significantly increased the expression level of TRPV4 channel in chondrocytes. The TRPV4 channel protein expression levels were normalized to GAPDH expression levels. Statistical results were from three independent experiments. (C) The mRNA expression level of the TRPV4 channel in chondrocytes was significantly higher on the stiff and medium substrates than on the soft substrate. Statistical results were from three independent experiments. GAPDH was used as a loading control. NS, not significant (P>0.05). *P<0.05, **P<0.01, ***P<0.001, respectively, compared with stiff substrate determined by one-way ANOVA and post hoc Tukey’s test.

Quantitative analysis of the TRPV4 fluorescence staining distribution showed that the relative fluorescence intensity for chondrocytes was significantly higher on the stiff substrate than on the soft substrate ( *P*<0.001; Figure 6A). Furthermore, stiff substrate significantly increased the TRPV4 protein and mRNA expression levels in chondrocytes ( *P*<0.001; Figure 6B,C). This suggested that substrate stiffness influences the TRPV4 channel protein level through regulating the TRPV4 transcriptional events. These results further supported our hypothesis that the TRPV4 channel is involved in the RVD response of chondrocytes sensing substrate stiffness.

## Discussion

In this study, we focused on the effects of substrate stiffness on the RVD response and calcium signaling in chondrocytes. Our results indicated that substrate stiffness-induced deformational discrepancies between the cell swelling and recovering under the hypo-osmotic stimulus. Soft substrate induced robust swelling of chondrocytes while stiff substrate contributed to a faster recovery behavior of chondrocytes during the RVD response ( Figure 2D,H). This is not surprising because chondrocytes on the stiff substrate have been shown to exhibit higher stiffness and lower cell height before the RVD response [19], which might make chondrocytes more difficult to swell in response to the hypo-osmotic stimulus. In contrast, the lower stiffness of chondrocytes on the soft substrate might make them easy to rapidly swell. But it is unclear yet why chondrocytes on the stiff substrate recover faster than the ones on the soft substrates. It is well known that stiff substrate induces cytoskeletal stiffening and hypo-osmotic challenge induces chromatin condensation, both of which contribute to cell stiffening [ 38, 41, 42]. Therefore, the higher increase in stiffness of chondrocytes on the stiff substrate may make them store higher cytoskeletal tension and more recovery energy. This would prevent chondrocytes from swelling but instead provide them with greater motility to recover. It has been suggested that cell stiffness decreases with increasing volume, as cells are grown on a substrate with fixed stiffness [43]. However, this is in contrast to our findings that the stiffness and viscoelasticity of chondrocytes on substrates of different stiffness are all increased at different degrees during the hypo-osmotic challenge. The differences may be caused by the cytoskeletal organization, the viscoelastic properties of the cell, and more importantly, the membrane rigidity. Thus, it is necessary to explore the regulation of substrate stiffness in the components of the cell membrane in articular chondrocytes, such as lipid and cholesterol [44]. We have also measured the mechanical properties of cells during the dynamic swelling process.

**Figure 7:**
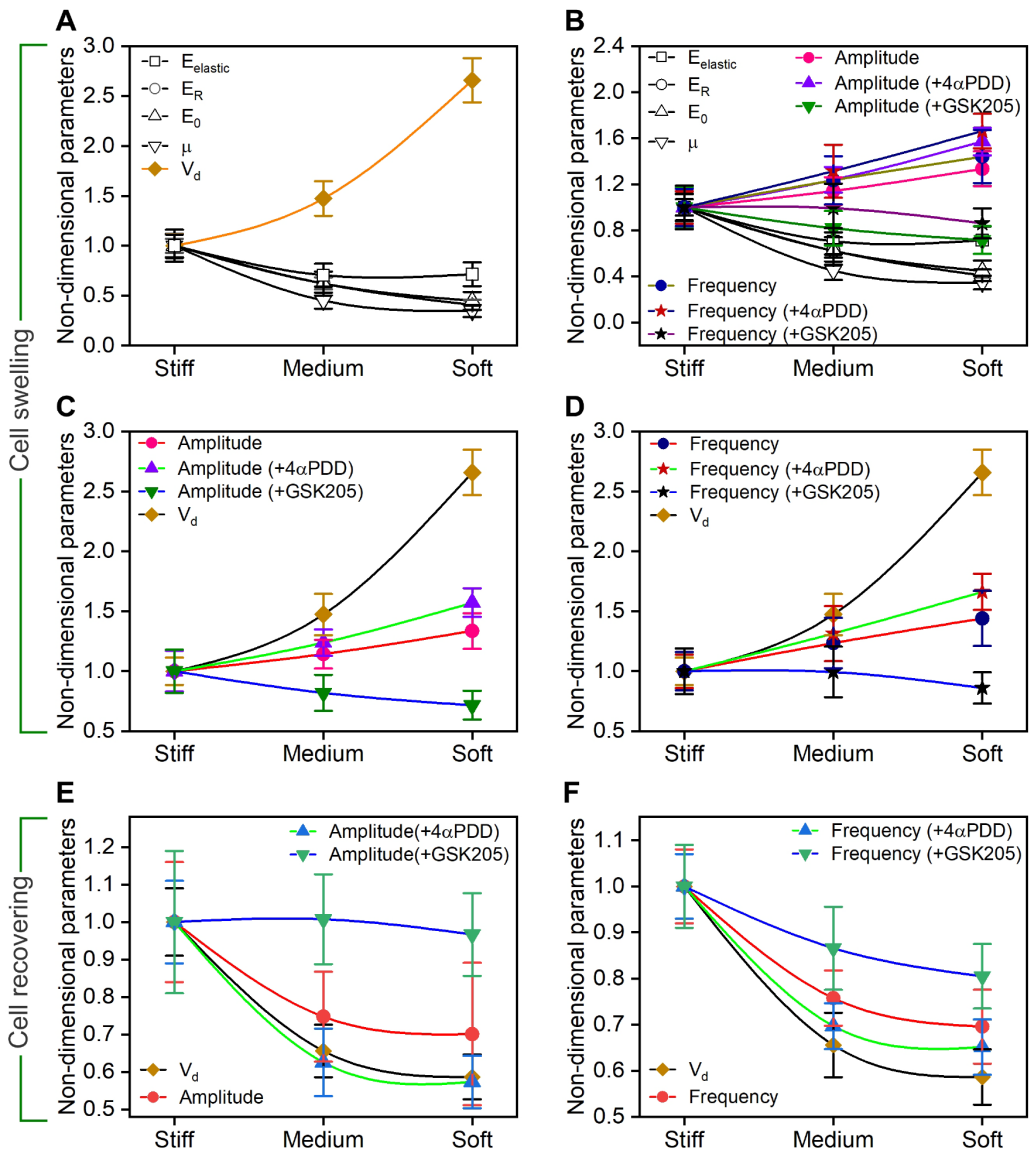
Figure7 **The relationship between non-dimensional mechanical parameters, RVD response parameters and cytosolic Ca ^2+^ oscillations parameters in chondrocytes on different substrates during cell swelling and recovering **(A) The non-dimensional parameter comparison between cell diameter rate Vd, and mechanical parameters, Eelastic, ER, E0 and μ during cell swelling on substrates of different stiffness. (B) The non-dimensional parameter comparison between the parameters of Ca2+ oscillations and the mechanical parameters, Eelastic, ER, E0 and μ during cell swelling on substrates of different stiffness. (C) The non-dimensional parameter comparison between cell diameter rate Vd, and the amplitude of Ca2+ oscillations during cell swelling on substrates of different stiffness. (D) The non-dimensional parameter comparison between cell diameter rate Vd, and the frequency of Ca2+ oscillations during cell swelling on substrates of different stiffness. (E) The non-dimensional parameter comparison between cell diameter rate Vd, and the amplitude of Ca2+ oscillations during cell recovering on substrates of different stiffness. (F) The non-dimensional parameter comparison between cell diameter rate Vd, and the frequency of Ca2+ oscillations during cell recovering on substrates of different stiffness.

Previous studies have focused on the inactive mechanical response of chondrocytes to external osmolarity or biochemical stimulus [ 38, 43– 46]. Under the two-dimensional (2D) substrate test conditions, the observed RVD response on substrates of different stiffness is directly related not only to the substrate stiffness-induced adhesive force but also to the applied hypo-osmotic challenge. Comparing the change trend of non-dimensional mechanical parameters, *E*_elastic_, *E*_R_ and *E*_0_ with cell diameter rate *V*_d_, we found that the *V*_d_ exhibited a reverse correlation with those substrate stiffness-dependent cell moduli during cell swelling on substrates ( Figure 7A). We also found that both the amplitude and frequency of Ca ^2+^ oscillations showed opposite trends with those parameters during cell swelling on different substrates ( Figure 7B).

Here, we showed that stiff substrate enhanced Ca ^2+^ oscillations of chondrocytes. Moreover, the Ca ^2+^ oscillations of chondrocytes in the hypo-osmotic medium were more robust compared with those in the iso-osmotic medium. This might be due to the large amounts of membrane area are involved in the membrane stretching during the hypo-osmotic challenge. However, this explanation is only suitable for the cell swelling process but not for the cell recovering process. So far, no mechanism underlying this phenomenon is available.

We found that the amplitude and frequency of Ca ^2+^ oscillations exhibited a consistent trend with the change in *V*_d_ during the cell swelling process on substrates with different stiffness ( Figure 7C,D). During the cell recovering process, the amplitude of Ca ^2+^ oscillations showed an inconsistent trend with the change in *V*_d_ ( Figure 7E), but the frequency of Ca ^2+^ oscillations showed a consistent trend with the change in *V*_d_ ( Figure 7F). This suggests that the correlation between the cell diameter rate *V*_d_ and Ca ^2+^ oscillations during the RVD response contributes to the mechanism underlying the RVD response. The observed difference in Ca ^2+^ oscillations of chondrocytes on different substrates may have important implications in regulating cell function through the cell-instructive substrate or scaffold. For example, an inappropriate increase in the chondrocyte volume leads to the progression of OA [20]. A robust Ca ^2+^ oscillations can enhance anabolic gene expression and matrix synthesis that are required to produce functional cartilage [30].

Our study further confirmed the hypothesis that Ca ^2+^ signaling is mediated by the TRPV4 channel, in a stiffness-dependent manner, in chondrocytes sensing substrate stiffness under iso-osmotic and hypo-osmotic conditions. However, TRPV4-mediated Ca ^2+^ oscillations of chondrocytes exhibited a more complex form during the RVD response induced by the hypo-osmotic challenge. For example, soft substrate significantly improved TRPV4-mediated Ca ^2+^ oscillations during cell swelling, which is consistent with a previous study showing that soft PCM increases the TRPV4-mediated Ca ^2+^ response to hypo-osmotic challenge [16]. On the other hand, stiff substrate greatly enhanced the TRPV4-mediated Ca ^2+^ oscillations during the cell recovering. It is puzzling that the regulation of substrate stiffness on calcium signaling of chondrocytes induced by the hypo-osmotic challenge is completely different from that induced by iso-osmotic challenge. Stiff substrate significantly enhanced the Ca ^2+^ oscillations of chondrocytes in the iso-osmotic medium ( Figure 4). This is consistent with the results that stiff substrate increases TRPV4 protein and mRNA expression levels in chondrocytes in the iso-osmotic medium ( Figure 6). This prompts us to think that the regulation mechanism of TRPV4 on calcium signals may be different under static and dynamic conditions. It is worth mentioning that *in vivo* PCM and other mechanical and biochemical factors might co-regulate Ca ^2+^ oscillations. Here, we only focus on the effects of 2D substrate stiffness on hypo-osmotic challenge-induced Ca ^2+^ oscillations of chondrocytes. However, our recent study indicated that Ca ^2+^ oscillations induced by a more physically-relevant 3D matrix are different from those by the 2D substrate [47]. Therefore, 3D cell-instructive substrate may help better understand the nature of the mechanical microenvironment of chondrocytes in the future studies [48].

It should be noted that substrate stiffness-induced cell stiffness may determine the capacity for cell volume regulation and TRPV4-mediated Ca ^2+^ oscillation. The TRPV4 channel functions as a volume sensor and is activated by increased cell volume, irrespective of the molecular mechanism underlying cell swelling [49]. However, the detailed regulatory mechanisms remain unknown. Therefore, the relationship between the rate of membrane deformation and calcium signaling during the cell swelling and recovering progress should be further examined. Additionally, the Ca ^2+^ oscillation in the native tissue remains to be determined because it is quite difficult to determine whether the impact of substrate stiffness on Ca ^2+^ signaling is chondroprotective or disease-promoting. Our work showed that substrate stiffness plays a critical role in regulating the RVD of chondrocytes and TRPV4-mediated mechanotransduction, though here we did not prove that substrate stiffness directly regulates the TRPV4 function. TRPV4 can be activated by a variety of other stimuli [50]. For example, cell swelling increases the membrane stretch and leads to the increase in contacts between the adhesive molecular and TRPV4, which activates TRPV4 channels by conformational change-associated ‘‘gate’’ opening through the actin reorganization [51]. But it is still challenging to demonstrate, *in vitro*, that the TRPV4 channel is directly gated by mechanical inputs [52].

In summary, our work highlights the importance of substrate stiffness as a physical cue of the microenvironment in modulating the RVD behavior. Furthermore, TRPV4-mediated Ca ^2+^ signaling of chondrocyte during the hypo-osmotic challenge depends on the substrate stiffness. Thus, RVD response and TRPV4-mediated Ca ^2+^ signaling, which are critical features of chondrocytes, may change in pathological conditions such as OA, thereby significantly affecting many cellular functions. In the future, it is necessary to explore the relationship between the calcium signaling and deformation rate in the cell swelling and recovering progress by direct membrane stretching experiments with different frequencies and magnitudes. It is well known that a 3D cell matrix that mimics key factors of tissue is much more representative of the *in vivo* microenvironment than a 2D substrate. Thus, with an increasing list of available three-dimensional cell-supporting biomaterials, investigating the roles of TRPV4 in regulating the chondrocyte RVD and calcium signal based on *in vivo* animal models will be more clinically significant. Our study on ion channels involved in the cell sensing matrix stiffness is beneficial for the design of cell-instructive scaffolds, which is crucial for chondrocytes to obtain appropriate mechanobiological behavior. Furthermore, the modulation of substrate stiffness-related mechanotransduction pathways, possibly by altering the TRPV4 function, may provide new therapeutic insights into the intervention of OA.

## Supporting information

21400Supplementary_Tables

## Supplementary Data

Supplementary data is available at *Acta Biochimica et Biophysica Sinica* online.
