# Supplementary material for: Substrate stiffness-dependent regulatory volume decrease and calcium signaling in chondrocytes: Substrate stiffness regulates chondrocyte volume and calcium signaling
Source: Acta Biochim Biophys Sin (Shanghai). 2021 Dec 16;54(1):113–25. doi: 10.3724/abbs.2021008 (PMC9909316; doi:10.3724/abbs.2021008)
Supplement: 21400Supplementary_Tables [file 21400Supplementary_Tables.docx]

**Supplementary Table S1. The RVD parameters of chondrocyte during swelling and recovering in hypo-osmotic medium (180 mOsm)**

| RVD responsive parameters | Stiff (*n*=29) | Medium (*n*=27) | Soft (*n*=32) |
| --- | --- | --- | --- |
| Initial diameter, d_0_ (μm) | 15 ± 3 | 13 ± 3 | 12 ± 2 |
| Maximum diameter, d_M_ (μm) | 19 ± 3 | 16 ± 2 | 16 ± 3 |
| Swelling time T_S_ (min) | 13 ± 2 | 9 ± 2 | 8 ± 1 |
| Percentage increase of d (%) | 22 ± 4 | 29 ± 3 | 33 ± 4 |
| Recovery time T_R_ (min) | 20 ± 4 | 31± 5 | 34 ± 4 |
| Percent of recovery diameter (%) | 75 ± 9 | 69 ± 6 | 61 ± 8 |
| Responding time T_Res_ (min) | 33 ± 4 | 40 ± 4 | 41 ± 4 |

*n* is the number of measured cells. All data are shown as the mean ± SD.

**Supplementary Table S2. The elastic and viscoelastic parameters of chondrocytes on varying substrate stiffness in iso-osmotic medium (320 mOsm)**

| Mechanical parameters | Stiff (*n*=32) | Medium (*n*=27) | Soft (*n*=36) |
| --- | --- | --- | --- |
| *E_elastic_* (kPa) | 1.6 ± 0.1 | 1.1± 0.2 | 1.0± 0.1 |
| *τ*_ε_ (s) | 4.5± 0.2 | 3.5± 0.2 | 3.2 ± 0.1 |
| *τ*_σ_ (s) | 7.4 ± 0.3 | 5.4 ± 0.3 | 5.1 ± 0.2 |
| *E*_R_ (kPa) | 0.5± 0.1 | 0.3 ± 0.1 | 0.2 ± 0.1 |
| *E*_0_ (kPa) | 0.8± 0.1 | 0.5 ± 0.1 | 0.4± 0.1 |
| *μ* (kPa⋅s) | 1.4± 0.1 | 0.7± 0.1 | 0.6± 0.1 |

*n* is the number of measured cells. All data are shown as the mean ±SD.

**Supplementary Table S3. The relative percentage increase of the mechanical parameters of chondrocytes on varying substrate stiffness in hypo-osmotic medium (180 mOsm) compared to the control group in iso-osmotic medium (320 mOsm)**

|  | *E_elastic_* (kPa) | *E*_R_ (kPa) | *E*_0_ (kPa) | *μ* (kPa⋅s) |
| --- | --- | --- | --- | --- |
| Stiff | 24% | 29% | 30% | 33% |
| Medium | 33% | 19% | 18% | 19% |
| Soft | 43% | 19% | 13% | 16% |

**Supplementary Table S4. The calcium responsive rate of chondrocytes on varying substrate stiffness induced by 4αPDD or GSK205 in iso-osmotic medium**

|  | Calcium responsive rate (%) | |
| --- | --- | --- |
|  | 4αPDD treated | GSK205 treated |
| Stiff | 80 ± 5 | 35 ± 4 |
| Medium | 54 ± 4 | 39 ± 3 |
| Soft | 53 ± 6 | 39 ± 4 |

All data are shown as the mean ± SD.

**Supplementary Table S**5. **The relative percentage increase or decrease of the amplitude and frequency of Ca^2+^ oscillations in chondrocytes on varying substrate stiffness induced by 4αPDD or GSK205 in iso-osmotic medium**

|  | 4αPDD- induced relative increase | | | GSK205- induced relative decrease | |
| --- | --- | --- | --- | --- | --- |
|  | Amplitude (ΔF/F_0_) | Frequency (peaks/min) | Amplitude (ΔF/F_0_) | | Frequency (peaks/min) |
| Stiff | 50% | 54% | 59% | | 52% |
| Medium | 44% | 43% | 46% | | 37% |
| Soft | 24% | 33% | 46% | | 24% |

**Supplementary Table S6. The percent of chondrocytes on variable stiffness substrates exhibited both RVD response and Ca^2+^ oscillations in hypo-osmotic (180 mOsm) medium after treatment of 4αPDD or GSK205**

|  | Calcium responsive rate (%) | |
| --- | --- | --- |
|  | 4αPDD treated | GSK205 treated |
| Stiff | 43 ± 5 | 24 ± 3 |
| Medium | 51 ± 5 | 29 ± 2 |
| Soft | 49 ± 5 | 33 ± 5 |

All data are shown as the mean ± SD.

**Supplementary Table S7. The relative percentage increase or decrease of the amplitude and frequency of Ca^2+^ oscillations in chondrocytes** **on varying substrate stiffness induced by 4αPDD or GSK205 during cells welling** **in hypo-osmotic (180 mOsm)**

|  | 4αPDD- induced relative increase | | GSK205- induced relative decrease | |
| --- | --- | --- | --- | --- |
|  | Amplitude (ΔF/F_0_) | Frequency (peaks/min) | Amplitude (ΔF/F_0_) | Frequency (peaks/min) |
| Stiff | 22% | 18% | 43% | 44% |
| Medium | 32% | 26% | 59% | 55% |
| Soft | 44% | 37% | 70% | 66% |

**Supplementary Table S8. The relative percentage increase or decrease of the amplitude and frequency of Ca^2+^ oscillations in chondrocytes on variable stiffness substrates induced by 4αPDD or GSK205 during cell recovering in hypo-osmotic (180 mOsm) medium**

|  | 4αPDD- induced relative increase | | GSK205- induced relative decrease | |
| --- | --- | --- | --- | --- |
|  | Amplitude (ΔF/F_0_) | Frequency (peaks/min) | Amplitude (ΔF/F_0_) | Frequency (peaks/min) |
| Stiff | 37% | 31% | 65% | 54% |
| Medium | 25% | 21% | 53% | 47% |
| Soft | 23% | 23% | 52% | 47% |
